# Impaired ankle inversion proprioception during walking is associated with fear of falling in older adults

**DOI:** 10.3389/fnagi.2022.946509

**Published:** 2022-09-30

**Authors:** Xuerong Shao, Zheng Wang, Lijiang Luan, Yilan Sheng, Ruoni Yu, Adrian Pranata, Roger Adams, Anren Zhang, Jia Han

**Affiliations:** ^1^Department of Rehabilitation Medicine, Shanghai Fourth People’s Hospital, School of Medicine, Tongji University, Shanghai, China; ^2^College of Rehabilitation Sciences, Shanghai University of Medicine and Health Sciences, Shanghai, China; ^3^School of Exercise and Health, Shanghai University of Sport, Shanghai, China; ^4^Department of Rehabilitation Medicine, Shanghai Sixth People’s Hospital Affiliated to Shanghai Jiao Tong University School of Medicine, Shanghai, China; ^5^School of Medicine, Jinhua Polytechnic, Jinhua, China; ^6^Faculty of Health, Arts and Design, Swinburne University of Technology, Hawthorn, VIC, Australia; ^7^Research Institute for Sports and Exercise, University of Canberra, Canberra, ACT, Australia

**Keywords:** ankle proprioception, fear of falling, falls, walking, elderly

## Abstract

**Background:**

Ankle proprioception plays a critical role in lower limb movement control. However, the relationship between ankle proprioception and fear of falling (FOF) in older people is still unclear.

**Objective:**

(1) This study aims to develop a new device for measuring ankle inversion proprioceptive discrimination sensitivity during walking, i.e., the Ankle Inversion Discrimination Apparatus–Walking (AIDAW), and assess the test–retest reliability of the AIDAW in both young and older adults; (2) to evaluate the discriminant validity of the measure by comparing ankle proprioception during walking between the two groups; and (3) to explore convergent validity by determining to what extent the AIDAW proprioceptive scores correlate with Fall Efficacy Scale-International (FES-I) scores.

**Materials and methods:**

The AIDAW was purpose-built to test ankle inversion proprioceptive discrimination sensitivity during walking. The area under the receiver operating curve (AUC) was calculated as the proprioceptive discrimination score. In total, 54 adults volunteered. Test–retest reliability was evaluated in 12 young and 12 older adults, and another 15 young and 15 older adults completed the comparison study. FOF was assessed by using the FES-I.

**Results:**

The test–retest reliability intraclass correlation coefficient ICC _(3,1)_ value for the whole group was 0.76 (95% CI: 0.52–0.89). The ICC values of the young and older groups were 0.81 (95% CI: 0.46–0.94) and 0.71 (95% CI: 0.26–0.91), respectively. The Minimal Detectable Change with 90% confidence (MDC_90_) values for the young and older groups were 0.03 and 0.11, respectively. There was a significant difference between the AIDAW proprioceptive sensitivity scores for the young and older groups (0.78 ± 0.04 vs. 0.72 ± 0.08, *F* = 5.06, *p* = 0.033). Spearman’s correlation analysis showed that the FES-I scores were significantly and negatively correlated with the AIDAW scores (*rho* = −0.61, *p* = 0.015), with higher FOF associated with worse ankle proprioception.

**Conclusion:**

The AIDAW is a reliable and valid device for measuring ankle proprioception during walking in both young and older adults. Ankle inversion proprioceptive discrimination sensitivity during walking was found to be impaired in the elderly compared to young adults. This impairment was found to be strongly associated with FOF, suggesting that assessment and intervention for ankle proprioception in this population are needed to reduce the risk of falls.

## Introduction

A decline in physical function is associated with aging, causing an increased risk of falling (Tieland et al., 2018). An estimated 684,000 people die from falls each year globally, of which adults over 60 suffer the highest number of fatal falls (World Health Organization, 2021). Falls in older adults may lead to severe injuries, such as lower limb fractures, resulting in disability, poor quality of life, or even death in older adults (Vaishya and Vaish, 2020). Therefore, fall prevention is critically important in an aging population.

Fear of falling (FOF) is among the most significant predictors of falls in the elderly (Jorgensen et al., 2017; Whipple et al., 2018). FOF is also harmful in the long term, resulting in restrictions on activities of daily life and reduced quality of life (Schoene et al., 2019). FOF seems to be as crucial in limiting the daily activities of older adults as multiple previous falls (Liu et al., 2021). It is strongly associated with a high incidence of falls in their daily activities and is one of the main predictors of falls in older adults (Schoene et al., 2019). Evidence suggests that physiological factors such as reduced muscle strength are essential in influencing the fear of falling (Yardimci et al., 2021). Thus, there is a need to understand the factors contributing to FOF, as commonly evaluated by the Fall Efficacy Scale-International (FES-I) (Delbaere et al., 2010).

Several studies have suggested that FOF is related to aging-related physical functional deficits. Specifically, a higher level of FOF is related to decreased dynamic balance control and muscle weakness (Kim et al., 2013; Park et al., 2014). Proprioception is an essential component in motor control. Research has shown that aging negatively affects proprioception, affecting the biomechanics of joints and the neuromuscular control of the lower limbs and leading to an increased likelihood of impaired balance and falls (Ferlinc et al., 2019). However, it is still unknown whether lower limb proprioception is related to FOF.

Previous studies (Han, 2013; Han et al., 2013) have shown that proprioceptive ability is joint-specific, which means that proprioception at different joints may contribute differently to balance control. Among all the lower limb joints, the ankle is arguably the most critical in lower limb balance control because the foot and ankle complex is the only part of the human body that comes in contact with the ground during gait (Han et al., 2015). Thus, investigating the relationship between ankle proprioception and FOF may provide helpful information in understanding the role of proprioceptive mechanisms in FOF.

In terms of methods for measuring ankle proprioception, there are three commonly used methods: threshold to detection of passive motion (TTDPM), joint position reproduction (JPR), and functional movement extent discrimination assessment (AMEDA) (Han et al., 2016). Han et al. (2021) argued that compared to the first two methods, the AMEDA has better ecological validity, as the test is conducted in full weight bearing, requiring active movement and with general vision and audition allowed during the test. The more function-like features of the AMEDA test may underlie its sensitivity to age-related changes. Yang et al. (2019) and Djajadikarta et al. (2020) used the AMEDA and TTDPM to assess proprioceptive change across the life span, results showed that only the AMEDA scores showed an ankle proprioceptive decline associated with aging.

Falls usually occur during walking (Hill et al., 1999). Based on the ecological validity concept of the AMEDA test methods, ankle proprioception should ideally be assessed during walking. For measuring proprioception during walking, some devices, such as a wearable robotized ankle-foot orthosis based on TTDPM test methods, have been shown to be a reliable and valid tool for assessing proprioception during walking (Fournier Belley et al., 2016; Dambreville et al., 2019; Bertrand-Charette et al., 2022). By delivering a trip-like perturbation during gait, this technological development has significantly improved the ecological validity of the TTDPM method. However, the weight of the device applied to the lower limb during walking may impact normal gait, and the additional weight may not be optimal for older adults, especially those with a higher risk of falling. In addition, the relationship between FOF and ankle proprioception during walking is still unknown.

Therefore, in our laboratory, we have developed a novel device to assess ankle proprioception during walking: the Ankle Inversion Discrimination Apparatus–Walking (AIDAW). The aims of this study were (1) to determine the test–retest reliability of the AIDAW for demonstrating between-subject variance and stability of AIDAW scores measured over time; (2) to assess the discriminant validity to verify whether the proprioceptive scores of the elderly are significantly different from those of the young; and (3) to evaluate convergent validity by determining whether AIDAW proprioceptive scores correlate with scores on the Fall Efficacy Scale-International (FES-I). The hypotheses of this study were that (1) the AIDAW would have good test–retest reliability; (2) ankle inversion proprioception during walking would be significantly lower in the elderly compared to young adults; and (3) proprioception scores in the elderly would be significantly related to their FES-I scores.

## Materials and methods

### Participants

Participants were recruited in the Shanghai Sport University and surrounding communities through posters and oral presentations from January to August 2020. From the study of Han et al. (2021), with power for reliability sample analysis of 0.80 with two observations per participant and the ICC estimated to be 0.50 with a significance level of 0.05 under the null hypothesis, the minimum required sample size of 22 was calculated. Finally, 24 participants were enrolled, including 12 young and 12 older adults (Han et al., 2021). The sample size for the validity study was calculated by analysis using the G*power software (Test family: *t*-tests; Statistical test: correlation (Point biserial model); power = 0.80; *p* = 0.05; effect size = 0.5). The minimum sample size needed for the validity study was 26. Twenty-four participants were recruited for the reliability study, including 12 young and 12 older adults. For the comparison study, another thirty participants were recruited for the discriminant and convergent validity studies, including 15 young and 15 older adults. Inclusion criteria were the young group aged 18–35 and the older group aged 65–80. The exclusion criteria were as follows: (1) lower extremity, spine, or head injury in the past 3 months (e.g., ankle sprain, fracture, and muscle strain); (2) visual or vestibular disorders; (3) neurological disorders (e.g., Parkinson’s disease, stroke, and multiple sclerosis); and (4) any other factors that may affect the results (e.g., having sport-specific training). All participants were right-footed, which was determined by the Chinese translation of the Waterloo footedness questionnaire (Revised) (Yang et al., 2018). All the tested foot in participants was right foot. Demographic information for the participants is shown in Table 1. The Human Ethics Committee approved the study at the Shanghai University of Sport (approval number: 102772020RT009), and all participants signed informed consent before data collection.

**TABLE 1:** Demographic characteristics of participants.

	Test–retest reliability study		Validity study	
	Young	Older	Young	Older
Participants, *n*	12	12	15	15
Male: Female, *n*	6:6	5:7	5:10	5:10
Age, year (Mean ± SD)	23.17 ± 1.40	69.36 ± 3.23	23.67 ± 1.95	70.60 ± 3.50
Weight, kg (Mean ± SD)	58.42 ± 7.59	59.31 ± 8.55	56.41 ± 7.44	60.05 ± 8.90
Height, m (Mean ± SD)	1.68 ± 0.06	1.57 ± 0.06	1.66 ± 0.07	1.58 ± 0.05

### Equipment

**FIGURE 1:**
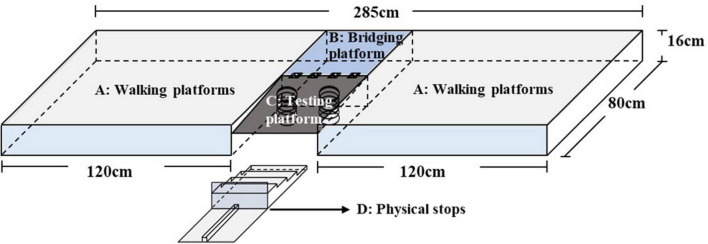
Depicts the components of the Ankle Inversion Discrimination Apparatus–Walking (AIDAW). **(A)**: Walking platforms; **(B)**: bridging platform; **(C)**: testing platform; and **(D)**: physical stops.

**FIGURE 2:**
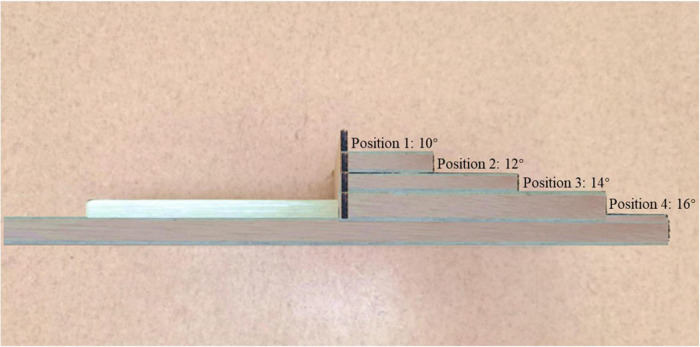
The lateral view of the physical stops generates ankle inversion angles of 10, 12, 14, and 16 degrees.

Based on signal detection theory for the calculation of the AUC measure (Zhang and Mueller, 2005), a new device—the Ankle Inversion Discrimination Apparatus–Walking (AIDAW, Figure 1)—was purpose-developed to assess ankle inversion movement discrimination sensitivity during walking, generating a measure of the accuracy of discrimination between angles of 10, 12, 14, and 16 degrees of inversion. The AIDAW consists of four components: (1) walking platforms (120 cm × 80 cm × 16 cm) for initiating gait before stepping across the testing platform and completing the gait cycle after the test; (2) a bridging platform (45 cm × 40 cm × 16 cm), which connects the walking and testing platforms; (3) a testing platform (45 cm × 42 cm × 1.2 cm), with two springs underneath to hold it in the same horizontal plane as the walking platforms; and (4) the physical stops, providing four ankle inversion degrees (Figure 2). During the test, when participants stepped onto the testing platform, it tilted to contact the movable physical stops, which generated the four predetermined ankle inversion positions: 1 = 10°, 2 = 12°, 3 = 14°, or 4 = 16°. The AIDAW is stable for walking on the testing platform in any four ankle inversion degrees, from 10 to 16°.

**FIGURE 3:**
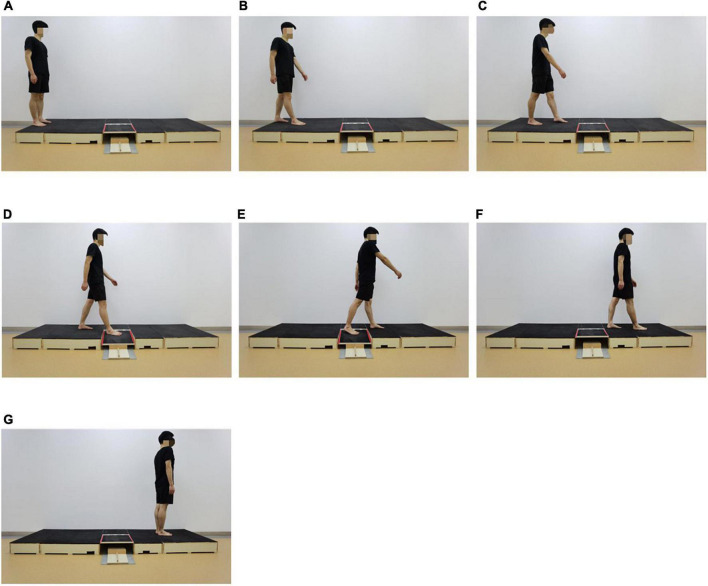
The AIDAW test. **(A)**: Starting position; **(B)**: Step 1; **(C)**: Step 2; **(D)**: Step 3; **(E)**: Step 4; **(F)**: Step 5; and **(G)**: Step 6.

The study was conducted in a proprioception research laboratory. Participants were instructed to stand upright, facing the walking platform with their eyes looking forward (Figure 3A). For each trial, participants were asked to walk normally for six steps on bare feet, with the testing foot to initiate a normal gait (Figure 3B) and the other foot to step onto the walking platform (Figure 3C), then step onto the testing platform (Figures 3D,E) until three full gait cycles were completed (Figures 3F,G). The order of the tilt angle of the testing platform was randomized. A valid trial included successful completion of the three gait cycles described and response regarding the inversion depth they perceived that they had just experienced. If participants did not step on the testing platform successfully but stepped on the junction between the walking platform and the testing platform, the test failed and then needed to be repeated.

Before data collection, participants had three rounds of standardized familiarization with the four ankle inversion positions in order (12 trials in total). During the test, each inversion angle was presented 10 times randomly, for 40 trials in total. The order of the randomized inversion angle in each trial was prepared in advance. Participants were required to respond to the ankle inversion position they had just experienced on each trial without any feedback as to the correctness of their responses. The response referred to the specific inversion angle they perceived at AIDAW during the test. The participants took 8–10 min to complete the AIDAW test.

### Questionnaire

The FES-I has good validity and reliability and has been recommended for research and clinical use (Delbaere et al., 2010). The scale contains 16 items scored on a 4-point scale, with points one representing not concerned, two somewhat concerned, three fairly concerned, and four very concerned. The higher the score, the more severe the fear of falling.

### Procedure

The study consisted of two parts. Part 1 was the test–retest reliability study, where the 24 participants were tested on two occasions by the same professional physiotherapist, 7 days apart. Part 2 was the comparison study, where the 30 participants completed the FES-I and the AIDAW tests in random order.

### Statistical analysis

**FIGURE 4:**
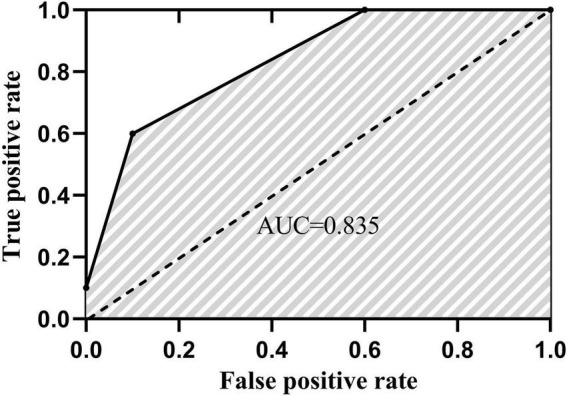
One participant example of the area under a ROC curve.

The pair-wised Receiver Operating Characteristic (ROC) curves of 40 ankle inversion stimuli and their corresponding pairs were produced by the non-parametric signal detection analysis by positions 1 and 2, 2 and 3, 3 and 4 (Han et al., 2013). The area under the ROC curve (AUC) was calculated as the ankle inversion proprioceptive discrimination score (AIDAW score). The demonstration figure of AUC is shown in Figure 4. The mean of the three pairs of wised AUC was calculated with SPSS. Test–retest reliability was indicated by Bland-Altman plots (Giavarina, 2015), and the Intraclass Correlation Coefficient, ICC _(3,1)_, with a two-way fixed model, single measure type, and absolute agreement definition, was calculated for test–retest reliability (Tao et al., 2021). With the result of ICC, the 90% CI was chosen for comparison with the Minimal Detectable Change (MDC) calculated with the Ankle Inversion Discrimination Apparatus for Landing (AIDAL) (Han et al., 2021). The Minimal Detectable Change with a 90% confidence interval (MDC_90_) was calculated using the formula (Steffen and Seney, 2008; Hulzinga et al., 2020) (*SEM*: the standard error; *s*: standard deviation of the measurements taken at the first time):

$$M\,D\,C_{90}=S\,E\,M\times 1.65\times \sqrt{2}$$

$$S\,E\,M=s\,\sqrt{1-I\,C\,C}$$

Given that the data for the older group were not normally distributed, the Mann–Whitney *U*-test was used to compare the difference in AIDAW scores between the young and the older groups (data shown as Median ± Interquartile Range).

The relationship between ankle inversion proprioceptive discrimination scores and FES-I scores was examined by Spearman’s correlation analysis (FES-I was the rank variable). All statistical analyses were performed using SPSS v24 (IBM Corporation Route 100, Somers, NY10589), with the significance level set at 0.05.

## Results

**FIGURE 5:**
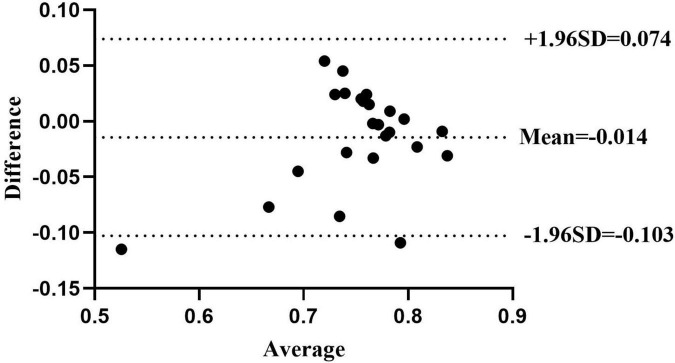
The Bland-Altman plot shows agreement between the older group’s first and second AIDAW tests. The mean difference score was –0.014, and the 95% limits of agreement were –0.103 and 0.074. All but two points fall within the 95% limits.

**FIGURE 6:**
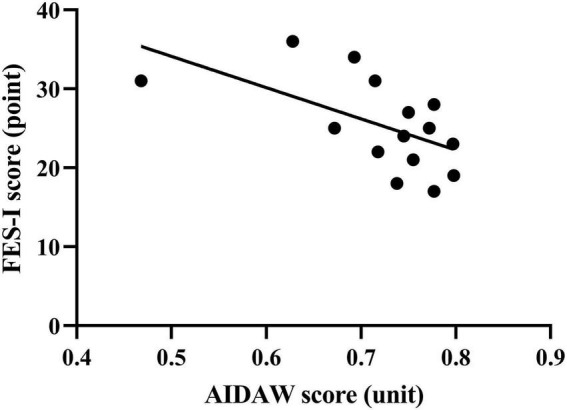
Correlation analysis of the AIDAW and FES-I scores in the older group (rho = –0.61).

The Bland-Altman plots for the whole group are shown in Figure 5. ICC _(3.1)_ values showed moderate-to-good test–retest reliability, with ICC _(3.1)_ = 0.76 in the whole group (95% CI: 0.52–0.89) (Koo and Li, 2016). Specifically, the ICC _(3.1)_ value was 0.81 for the young group (95% CI: 0.46–0.94) and 0.71 for the older group (95% CI: 0.25–0.91). The MDC_90_ scores for the young and older groups were 0.03 and 0.11. The AIDAW scores in the young group were significantly lower than those in the older group (0.77 ± 0.07 vs. 0.75 ± 0.08, *Z* = 2.013, *p* = 0.044). Using Spearman’s correlation, we showed that FES-I scores were significantly and moderately negatively correlated with the AIDAW scores (*rho* = −0.61, *p* = 0.015, Figure 6) (Schober et al., 2018).

## Discussion

In the present study, an apparatus for measuring ankle inversion proprioception during walking has been developed, and its test–retest reliability and validity have been examined. Previous studies have developed a wearable ankle-foot device for assessing ankle proprioception associated with perturbations applied during walking, based on the TTDPM method (Fournier Belley et al., 2016; Dambreville et al., 2019; Bertrand-Charette et al., 2022). The current apparatus was developed based on the AMEDA method of assessing the ability to discriminate between different angles of inversion movement extent (Han, 2013; Han et al., 2016, 2021). The Bland-Altman plots and ICC _(3.1)_ showed that the AIDAW had moderate-to-good test–retest reliability in the combined young, older, and the whole group. In parallel with a previous somatosensory apparatus, the Ankle Inversion Discrimination Apparatus for Landing (AIDAL), which was recently developed for testing ankle inversion proprioceptive discrimination during landing, and reliability testing showed a moderate-to-good test–retest reliability in the CAI group and non-CAI groups (ICC = 0.701 and ICC = 0.804, respectively) (Han et al., 2021). In addition, the sway discrimination apparatus (SwayDA) was developed to assess voluntary postural sway proprioceptive discrimination sensitivity and showed ICCs of 0.750 and 0.879 for left and right postural sway discrimination, respectively (Chen et al., 2019). Compared to previous proprioception testing methods, JPS or TTDPM have shown superior test–retest reliability (Bertrand-Charette et al., 2020), but at values consistent with the present study’s results.

The MDC_90_ values generated from this study would be useful references to determine meaningful clinical changes (Donoghue et al., 2019). In the current study, the MDC_90_ for the young group was 0.031, which was similar to the AIDAL findings (Han et al., 2021). In contrast, the MDC90 for the older group was larger than the young group, suggesting greater test–retest variability, such that the AIDAW has a larger measurement error when used for detecting real change that would reflect the effectiveness of an intervention program in this group. From its computational formula, MDC is negatively influenced by a low ICC value. Given that the ICC for the older group was relatively low, the MDC for this group was larger than the younger group. This finding suggests that older adults may have greater variability in their ankle proprioceptive sensitivity. Other sensorimotor research has found that older adults show greater motor output variability in ankle movement control than their younger counterparts (Lodha et al., 2016). Therefore, interventions that target ankle proprioception in older people must consider the larger MDC value so as to be able to confirm a true and clinically important change.

The discriminant validity study showed that ankle proprioception in older people during walking was significantly worse than that of young people. This finding was consistent with Yang et al. (2019), who found poorer ankle proprioception in older than young people when tested using the AMEDA while standing in full weight bearing. In contrast, the TTDPM method did not reveal any difference in passive movement ankle proprioception between young and older adults (Djajadikarta et al., 2020). For JPS, Franco et al. (2015) compared the performance of young and elderly participants in terms of position sense of the ankle and hip joints, and no significant differences were found. These discrepancies may be due to the different methods used. Han et al. (2021) argued that the active movements used in the AMEDA test allowed the brain to integrate vestibular and proprioceptive information to discriminate ankle movement extents. Thus, the AMEDA method tested “obtained proprioception” while the TTDPM method tested the ability to perceive movement passively imposed on a body segment (Weerakkody et al., 2008), which is “imposed proprioception” (Han et al., 2020). These findings suggest that the AMEDA and AIDAW methods may better examine the central mechanisms underlying proprioceptive impairments associated with the aging-neural noise hypothesis (Henry and Baudry, 2019). Aging is associated with alterations to muscle spindles and their neural pathways, which may contribute to the lower signal-to-noise ratio that challenges the integration of proprioceptive signals. On the one hand, the ability of the central nervous system to process neural noise decreases with age. This notion is supported by neuroimaging studies, where it has been found that aging leads to a decline in right putamen activation in the central processing of proprioceptive information (Goble et al., 2012).

For convergent validity, with regard to the negative correlation relationship between AIDAW and FES-I scores, these test scores are the first to establish an association between an ankle proprioceptive measure, AIDAW scores, and FOF, suggesting that the worse the ankle proprioception, the higher the level of FOF in older people. Indeed, this association reflects a degree of realism among older people with deficits. The study by Waddington and Adams (2003) showed that even 0.04° of increased inversion uncertainty potentially increases the frequency of falling from 1.2 to 1.22%. Although this 0.02% increase in the frequency of falling seems small, considering the large number of gait cycles undertaken in daily activities, it may become an important factor in contributing to injury incidence. Therefore, although the difference in proprioception between the two groups in this study does not appear to be large, it has important implications for ankle proprioception and may increase the risk of falling in older adults. In a previous study, Toosizadeh et al. (2018) also found proprioceptive deficits among high-fall-risk individuals compared to healthy participants when balance performance was disturbed using low-frequency mechanical calf vibration.

Further studies could examine the association between ankle proprioception and falls and high FOF and actual falls. In addition, previous research has shown that a higher level of FOF is related to decreased balance and muscle weakness (Kim et al., 2013; Park et al., 2014). Therefore, the present finding extends these results to add ankle proprioceptive deficits into the equation, indicating the importance of assessment and intervention targeting ankle proprioception in this population. Researchers have found that active interventions such as exercise could reduce FOF by a small to moderate degree for older people after immediate exercise intervention (Kumar et al., 2016). Similarly, a systematic review reported that tai chi, walking, and water-based exercise that aimed to improve strength and balance ability could all reduce the level of FOF (Whipple et al., 2018). Future research needs to determine what types of intervention would improve ankle proprioception during walking. Previously, Martínez-Amat et al. (2013) found that 12 weeks of proprioception training could improve postural stability, static and dynamic balance, and gait in the elderly. Waddington and Adams (2004) found that a 5-week home-based wobble board training effectively enhanced ankle inversion proprioception measured in standing. Therefore, passive and active interventions that target improving ankle proprioception during walking should be considered for physiotherapy programs designed to lower the level of FOF and reduce the risk of falls in older people.

## Strengths and limitations

This study included both young and old people and found significant differences in proprioception during walking between the two groups, which is an important finding for understanding the relationship between age and proprioceptive changes. In addition, we have provided MDC values for the AIDAW in both populations and suggested that interventions targeting ankle proprioception in different populations must consider the MDC value to be able to confirm a true and clinically important change. Moreover, this study found that proprioception during walking is closely related to fear of falling. This result has important clinical implications for interventions to prevent falls in older adults.

Compared to other methods that rely on advanced technologies (Fournier Belley et al., 2016; Dambreville et al., 2019; Bertrand-Charette et al., 2022), the current AIDAW apparatus is easier to build, from inexpensive materials, and does not require any attachment to the body. In addition, only one examiner, rather than a research team, is needed to complete the assessment, and the whole assessment takes less than 15 min. All these features make the assessment a feasible one for clinical settings.

Fatigue levels should be measured and recorded for older adults before and after the AIDAW test, as the older participants are particularly vulnerable to fatigue. In addition, other data could be gathered for this study. Walking speed, stride length, and some gait parameters may be beneficial to studying the differences in walking in older groups in future research. In this study, we only used the AIDAW scores and FES-I data. Although they can reflect psychological problems, neurological changes are still unknown. To better understand the influence of various diseases on clinical proprioceptive testing, future research could compare the ankle proprioception of different groups of older individuals while walking using the AIDAW. There is also space consideration regarding the apparatus. As the AIDAW covers an area of 3 by 1 meters, the tester needs access to a large space for using the AIDAW, and clinical use of the AIDAW may therefore take some time to promote.

## Conclusion

The AIDAW is a novel, reliable, and valid device for assessing ankle inversion proprioceptive discrimination sensitivity during walking in both young and older adults. Ankle inversion proprioceptive discrimination sensitivity of older people was found to be impaired, and ankle inversion proprioceptive discrimination sensitivity during walking was significantly associated with FOF in older adults. Therefore, the AIDAW proprioception assessment system, or any system that could reflect ankle proprioception in functional walking, should be considered in physiotherapy assessment and intervention to improve symptoms and reduce the risk of falls in older adults.

## Data availability statement

The raw data supporting the conclusions of this article will be made available by the authors, without undue reservation.

## Ethics statement

The studies involving human participants were reviewed and approved by the Human Ethics Committee of Shanghai University of Sport. The patients/participants provided their written informed consent to participate in this study.

## Author contributions

XS collected the data, analyzed the data, and drafted the manuscript. ZW collected the data and edited the manuscript. LL, YS, RY, AP, and RA helped to edit the manuscript. AZ and JH designed the study, helped to analyze the data, and edited the manuscript. All authors contributed to the article and approved the submitted version.
